# Transcriptomes and expression profiling of deep-sea corals from the Red Sea provide insight into the biology of azooxanthellate corals

**DOI:** 10.1038/s41598-017-05572-x

**Published:** 2017-07-25

**Authors:** Lauren K. Yum, Sebastian Baumgarten, Till Röthig, Cornelia Roder, Anna Roik, Craig Michell, Christian R. Voolstra

**Affiliations:** Red Sea Research Center, Division of Biological and Environmental Science and Engineering (BESE), King Abdullah University of Science and Technology (KAUST), Thuwal, 23955-6900 Saudi Arabia

## Abstract

Despite the importance of deep-sea corals, our current understanding of their ecology and evolution is limited due to difficulties in sampling and studying deep-sea environments. Moreover, a recent re-evaluation of habitat limitations has been suggested after characterization of deep-sea corals in the Red Sea, where they live at temperatures of above 20 °C at low oxygen concentrations. To gain further insight into the biology of deep-sea corals, we produced reference transcriptomes and studied gene expression of three deep-sea coral species from the Red Sea, i.e. *Dendrophyllia* sp., *Eguchipsammia fistula*, and *Rhizotrochus typus*. Our analyses suggest that deep-sea coral employ mitochondrial hypometabolism and anaerobic glycolysis to manage low oxygen conditions present in the Red Sea. Notably, we found expression of genes related to surface cilia motion that presumably enhance small particle transport rates in the oligotrophic deep-sea environment. This is the first study to characterize transcriptomes and *in situ* gene expression for deep-sea corals. Our work offers several mechanisms by which deep-sea corals might cope with the distinct environmental conditions present in the Red Sea As such, our data provide direction for future research and further insight to organismal response of deep-sea coral to environmental change and ocean warming.

## Introduction

The importance of deep-sea corals and the reefs they build is becoming increasingly evident^1, 2^. Deep-sea reefs constitute biodiversity hotspots with micro-niches for many species^1^. Indeed, over 1,300 metazoan species have been documented on a single deep-sea coral *Lophelia pertusa* reef, in the northeast Atlantic Ocean^2^. The habitats of deep-sea coral occur commonly at depths of greater than 200 m (although they are also present at shallower depths at high latitudes) and can be found in the cold, dark, nutrient-rich water masses in nearly all of the world’s oceans, e.g. in fjords, along the edges of continental shelves, and around offshore submarine banks^2^. Deep-sea coral, in comparison to their shallow-water counterparts, have no symbiotic algae to provide energy, but rather have to rely on capture-feeding of particulate organic matter and zooplankton^1, 3^.

The principal ecological aspects of deep-sea corals, including the habitat’s environmental conditions, are only beginning to be understood. Until recently, deep-sea corals were synonymous with cold-water corals, as they were only observed at sites featuring temperatures between 4 to 14 °C^1^. However, Roder, *et al*.^4^ and Qurban, *et al*.^5^ described deep-sea coral reefs in the Red Sea at much higher temperatures (>20 °C). Similarly, salinity measurements near deep-sea reefs commonly range from 32–38.8 PSU^1^, whereas salinity levels in the Red Sea typically exceed 40 PSU^4^. Further, the lowest dissolved oxygen (DO) limit where *L*. *pertusa*, a cosmopolitan azooxanthellate coral, occurs, has been measured at 2.6 mg/l in the Gulf of Mexico^6^. In contrast, lowest measured oxygen concentrations for deep-sea corals from the Red Sea were below 1 mg/l^4^. Thus, deep-sea corals in the Red Sea live in markedly different conditions from deep-sea corals elsewhere.

At least seven distinct calcifying deep-sea coral species have been observed in the Red Sea so far^4, 5^. Interestingly, *Rhizotrochus typus and Eguchipsammia fistula*, two of the species identified in the Red Sea, are not Red Sea endemics. *R*. *typus* also occurs in the deep-sea of the Indo-West Pacific and *E*. *fistula* is a cosmopolitan coral that is also found in the deep seas of the IndoPacific and Oceania^7^. Collected specimens of *E*. *fistula* from the Red Sea showed extreme tissue reduction, where individual corals were rarely covered by tissue below the growing edge of the polyps and different polyps were rarely connected by tissue^4^. Accordingly, this was offered as one possible adaptation to reduce metabolic demands in the oligotrophic, warm, and oxygen-depleted Red Sea deep-sea environment^4^. At the same time, this adjustment might represent a case of extreme phenotypic plasticity, as regularly fed aquaria-reared coral specimens of *E*. *fistula* from the Red Sea displayed substantial tissue re-growth over the entire skeleton^8^.

The common difficulty of accessing deep-sea corals dictates that the majority of experiments performed on these corals must be done *ex situ*, which might bias physiological responses and derived measurements. Further, the majority of deep-sea coral studies have been conducted on one particular species, *Lophelia pertusa*, due to its global distribution and its presence at relatively shallow depths, facilitating accessibility and comparative analyses^6, 9, 10^. While the genome of *L*. *pertusa* is currently being sequenced^11^, transcriptome and gene expression data for cold-water corals are missing to date at large. To further understand the ecology and physiology of deep-sea corals and extend the catalog of deep-sea coral species for which molecular data are available, we conducted transcriptome sequencing and gene expression analysis of three species of deep-sea corals, i.e. *Dendrophyllia* sp., *Eguchipsammia fistula*, and *Rhizotrochus typus*, collected in the central and northern Red Sea. Availability of the first transcriptomes and gene expression data for deep-sea corals might yield important insight in regard to genomic content and the mechanisms that corals employ to cope with the distinct environmental conditions of the Red Sea and deep-sea environments at large. Further, analysis of the cosmopolitan *E*. *fistula* collected from the warm Red Sea might provide a baseline for further research to the response of deep-sea corals to environmental change and ocean warming.

## Results

In this study we produced reference transcriptomes for three deep-sea coral species, *Dendrophyllia* sp., *E*. *fistula*, and *R*. *typus* (collected at depths of 625–630 m, 314–320 m, and 970–993 m, respectively) from the central and northern Red Sea in order to gain insight into the genes and pathways present in deep-sea corals. In addition, we assessed and compared gene expression for the three coral species in order to elucidate their physiology and the mechanisms these species employ to cope in a deep-sea environment. By preserving coral specimens for *E*. *fistula* and *R*. *typus* at depth, our data should provide a snapshot of *in situ* gene expression of deep-sea corals in their natural environment.

### Deep-sea coral transcriptomes

**Figure 1:**
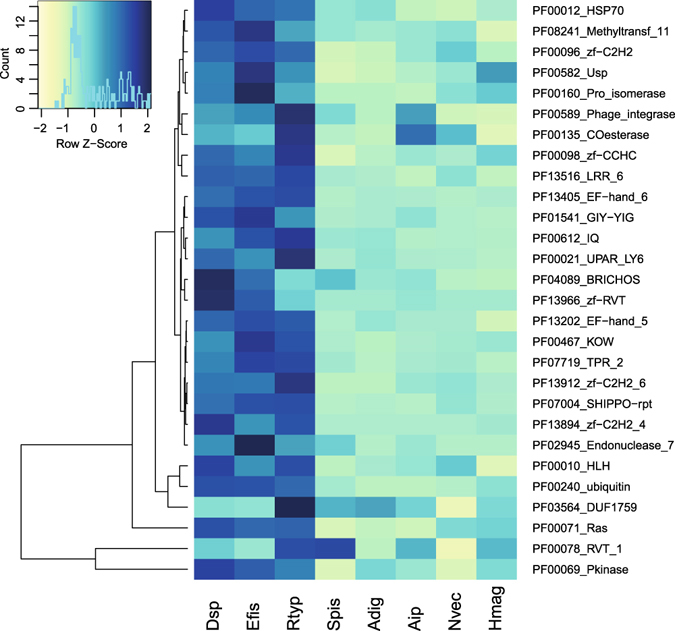
Enrichment of Pfam-based protein domains of three deep-sea coral transcriptomes in comparison to genomic gene sets of other cnidarians. Rows depict Z-score transformed Pfam domain counts and columns represent organisms. Only significantly enriched domains in deep-sea corals are displayed (FDR < 0.05). Rows are clustered by Euclidean distance. Color key indicates domain counts over row z-scores alongside distribution (histogram). Dsp = *Dendrophyllia* sp., Efis = *Eguchipsammia fistula*, Rtyp = *R*. *typus*, Spis = *Stylophora pistillata*, Adig = *Acropora digitifera*, Aip = *Aiptasia*, Nvec = *Nematostella vectensis*, Hmag = *Hydra magnipapillata*.

Transcriptomes of the three coral species, *Dendrophyllia* sp., *E*. *fistula*, and *R*. *typus*, consisted of 43,748, 36,545, and 41,186 assembled gene loci, respectively. Of these, 21,337, 16,609, and 21,123 genes were annotated with SwissProt/TrEMBL, and 17,689, 13,795, and 17,065 genes had GO annotations (Table 1). We were interested to understand whether deep-sea coral transcriptomes encode for a similar functional content as their shallow-water symbiotic counterparts and other cnidarians. For this reason, we annotated the protein-translated transcriptomic gene sets based on protein domains and subsequently tested for enrichment against the domain-annotated protein-translated genomic gene sets of other cnidarian genomes, i.e. *Stylophora pistillata*, *Acropora digitifera*, *Aiptasia* sp., *Nematostella vectensis*, and *Hydra magnipapillata* (Fig. 1). A total of 5,175 domains were annotated and present at least once in any of the species. Of these, 28 domains were significantly enriched in at least one deep-sea coral transcriptome. Interestingly, domain enrichment was remarkably conserved across deep-sea corals and distinct from cnidarian genomic gene sets indicating that deep-sea coral have a common set of enriched functions. The enriched domains encoded for DNA binding domains, in particular Zinc finger domains (PF00096_zf-C2H2, PF13894_zf-C2H2_4, PF00098_zf-CCHC, PF13912_zf-C2H2_6) and helix loop helix domains (PF00010_HLH). Further, we found enrichment of Ca^2+^-binding and -sensing domains (PF13405_EF-hand_6, PF13202_EF-hand_5, PF00612_IQ), reverse transcriptase-related and phage-associated domains (PF00589_Phage_integrase, PF13966_zf-RVT, PF00078_RVT_1), and domains related to stress response (PF00012_HSP70, PF00582_Usp).

**Table 1:** Sequencing statistics and transcriptome assembly statistics for three deep-sea coral species from the Red Sea represented by four colonies each.

Species	Read pairs (mio)	Reads retained (%)	assembled bp (mio)	assembled genes	annot. genes	annot. genes (%)	annot. genes (GO)	contig N50 (bp)	GC (%)
*Dendrophyllia* sp. 1	21.9	93.75	133	43,748	21,337	48.77	17,689	1,717	41.85
*Dendrophyllia* sp. 2	12.6	93.88							
*Dendrophyllia* sp. 3	16.2	94.14							
*Dendrophyllia* sp. 4	16.6	94.23							
*E*. *fistula* 1	11.2	93.41	90.8	36,545	16,609	45.45	13,795	1,396	41.11
*E*. *fistula* 2	12.3	92.92							
*E*. *fistula* 3	14.6	93.35							
*E*. *fistula* 4	15.7	93.09							
*R*. *typus* 1	12.4	94.16	131	41,186	21,123	51.29	17,065	1,805	41.88
*R*. *typus* 2	15.7	93.77							
*R*. *typus* 3	13.8	93.59							
*R*. *typus* 4	17.9	92.2

### Highly expressed genes of deep-sea corals

**Figure 2:**
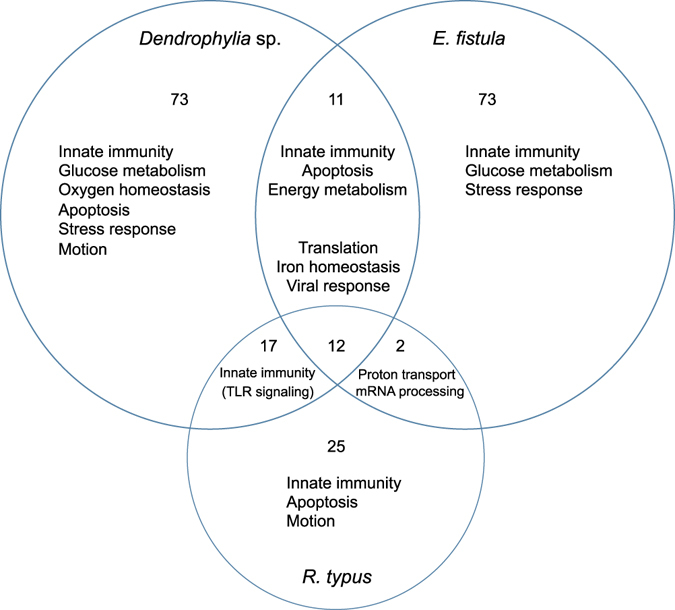
Overview of selected enriched GO biological process terms for highly expressed genes (FPKM > 100). Values represent the number of shared and exclusive GO terms of three deep-sea coral species from the Red Sea.

We selected a set of highly expressed genes to further understand the prevailing expressed functions and pathways. Highly expressed genes were defined as those genes with an FPKM (Fragments Per Kilobase of transcript per Million mapped reads) value > 100 (averaged over replicates of a given species). This resulted in 832, 750, and 466 genes that were highly expressed, of which 545, 543, and 338 were annotated in *Dendrophyllia* sp., *E*. *fistula*, and *R*. *typus*, 477, 486, and 302 of which had GO annotations, respectively (Supplemental Information, Dataset S1). Analysis of these genes revealed 113, 98, and 56 enriched GO terms annotated under the biological process ontology for *Dendrophyllia* sp., *E*. *fistula*, and *R*. *typus*, respectively (Fig. 2). The majority of GO terms were distinct for each species, but we identified 12 significantly enriched GO terms common to all three deep-sea coral species. These terms could be assigned to housekeeping processes such as cell cycle (mitotic spindle elongation, centrosome duplication), translation (cytoplasmic translation, translation, translational initiation, translational elongation, translational termination), or transcription (positive regulation of nuclear-transcribed mRNA poly(A) tail shortening, positive regulation of nuclear-transcribed mRNA catabolic process, deadenylation-dependent decay). We also found shared processes that were more specific and are putatively related to the deep-sea habitat, e.g. ‘cellular iron ion homeostasis’ and ‘viral transcription’ (Fig. 2). Interestingly, *Dendrophyllia* sp. and *R*. *typus* shared 17 terms almost exclusively related to Toll-like receptor (TLR) signaling pathways (MyD88-dependent Toll-like receptor signaling pathway, Toll-like receptor 2, 3, 4, 5, 7, 15, and 21), which are known to play a key role in the innate immune system^12, 13^. The majority of enriched GO terms were, however, species-specific. *Dendrophylia* sp. had 73, *E*. *fistula* 73, and *R*. *typus* 25 non-shared enriched GO terms within the set of highly expressed genes (Fig. 2). Manual sorting of the enriched GO terms revealed that all corals highly expressed genes that could be related to the immune system and motion (Table 2). Other enriched GO terms in *Dendrophyllia* sp. could be assorted to processes related to glucose and oxygen homeostasis. *E*. *fistula* had several enriched GO terms involved in cell-cell interaction. *R*. *typus* had overall fewer enriched GO terms compared to the other two species, but many associated with sensory and cell-matrix interaction.

**Table 2:** Significantly enriched GO terms for highly expressed genes (FPKM > 100) for three deep-sea coral species from the Red Sea (*P* value < 0.05), manually sorted into higher order categories.

	GO ID	Term	*P* value
***Dendrophyllia*** **sp**.			
Immune system	GO:0002821	pos. reg. of adaptive immune response	0.02651
	GO:0034097	response to cytokine stimulus	0.02712
	GO:0070243	reg. of thymocyte apoptotic process	0.02742
	GO:0001782	B cell homeostasis	0.03293
Motion	GO:0051017	actin filament bundle assembly	0.00683
	GO:0030048	actin filament-based movement	0.01306
	GO:0051016	barbed-end actin filament capping	0.02742
Glucose Response	GO:0042149	cellular response to glucose starvation	0.03884
	GO:0001678	cellular glucose homeostasis	0.01347
	GO:0006111	reg. of gluconeogenesis	0.03293
	GO:0006096	glycolysis	0.03749
Oxygen	GO:0002931	response to ischemia	0.01767
	GO:0071456	cellular response to hypoxia	0.00187
	GO:0032364	oxygen homeostasis	0.01767
***E***. ***fistula***			
Immune system	GO:0002686	neg. reg. of leukocyte migration	0.00077
	GO:0032088	neg. reg. of NF-kappaB transcription factor activity	0.00525
	GO:0002861	reg. of inflammatory response to antigenic stimulus	0.01122
	GO:0032649	reg. of interferon-gamma production	0.0164
	GO:0032480	neg. reg. of type I interferon production	0.02933
	GO:0002891	pos. reg. of immunoglobulin mediated immune response	0.03467
	GO:0002922	pos. reg. of humoral immune response	0.03467
	GO:0006958	complement activation, classical pathway	0.03478
Motion	GO:0042989	sequestering of actin monomers	0.01645
	GO:0060088	auditory receptor cell stereocilium organization	0.02251
	GO:0032528	microvillus organization	0.01122
	GO:0072673	lamellipodium morphogenesis	0.03686
Cell-cell interaction	GO:0010560	pos. reg. of glycoprotein biosynthetic process	0.00077
	GO:0071554	cell wall organization or biogenesis	0.00687
	GO:0030155	reg. of cell adhesion	0.01621
***R***. ***typus***			
Immune system	GO:0045408	reg. of interleukin-6 biosynthetic process	0.01386
Motion	GO:0045475	locomotor rhythm	0.00553
	GO:0051014	actin filament severing	0.01386
Sensory	GO:0043207	response to external biotic stimulus	0.01139
Cell-matrix interaction	GO:0022617	extracellular matrix disassembly	0.02896
	GO:0030199	collagen fibril organization	0.03914
	GO:0007016	cytoskeletal anchoring at plasma membrane	0.04744
	GO:0007160	cell-matrix adhesion	0.04794

### Deep-sea coral orthologs and differential gene expression

In order to compare differences between all three deep-sea coral species, we identified orthologs and conducted a differential gene expression analysis *sensu* Parkinson, *et al*.^14^. Of the 23,280 (*Dendrophyllia sp*.), 17,376 (*E*. *fistula*), and 22,025 (*R*. *typus*) ORFs (open reading frames) determined, 6,713, 7,501, and 5,497 pairwise orthologs were identified for the species pairs *Dendrophyllia* sp. - *E*. *fistula*, *Dendrophyllia* sp. - *R*. *typus*, and *E*. *fistula* - *R*. *typus*, respectively. Of these, 2,069 orthologs were identified in all three taxa. A comparison between expression profiles across the 2,069 orthologs showed substantial expression differences between species as indicated by a principal component analysis, in which almost 80% of the differences were explained in the first two components that separated all species from each other (Supplemental Information, Figure S1).

**Figure 3:**
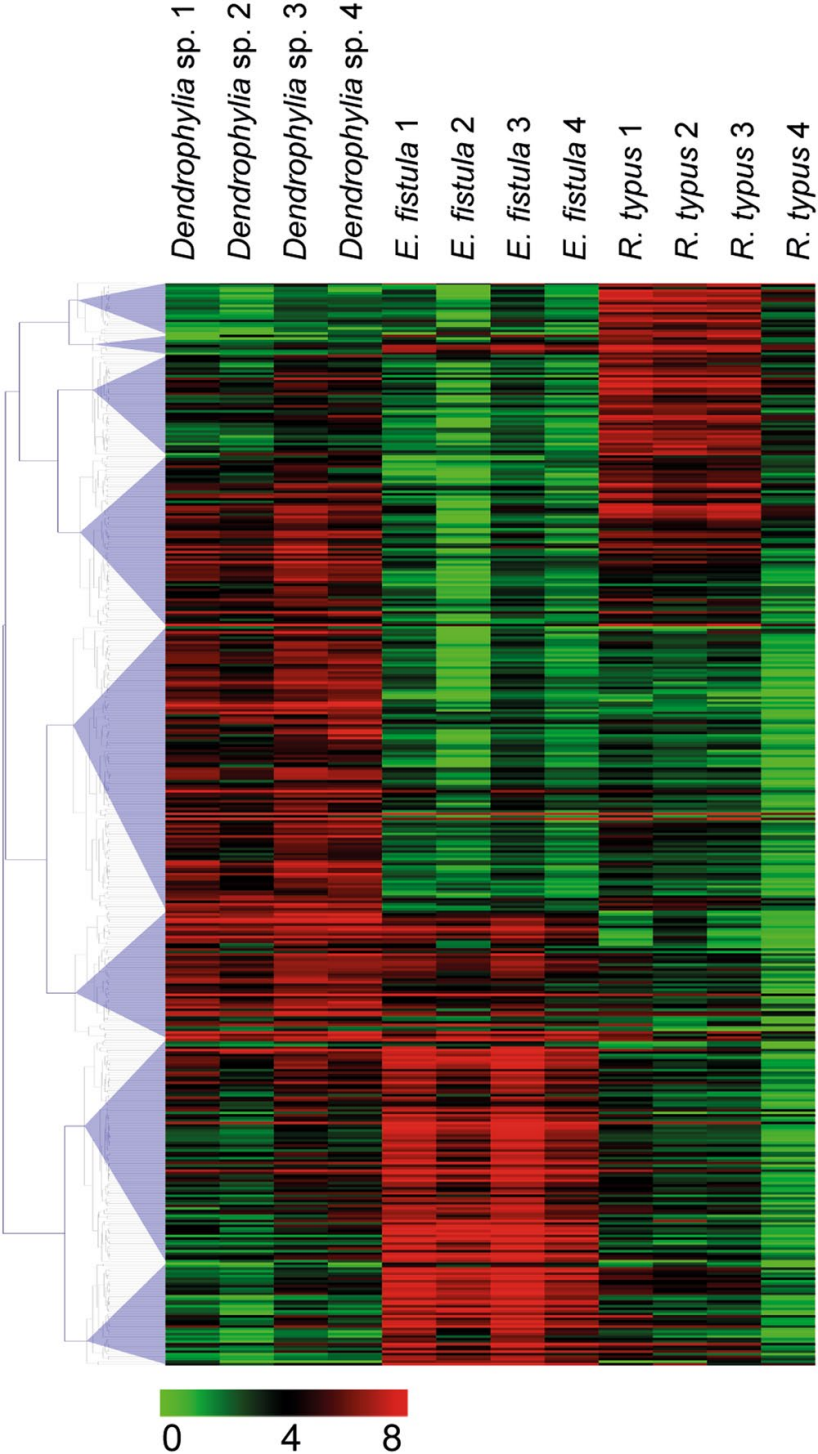
Heatmap of 431 differentially expressed orthologous genes between *Dendrophyllia* sp., *E*. *fistula*, and *R*. *typus*. Colors denote log_2_(x + 1) transformed FPKM values.

We found significant gene expression differences in 431 orthologs (20.8%) that clustered into 8 groups (Fig. 3). Among the differentially expressed genes, a homolog of the hypoxia inducible factor was notably higher expressed in *Dendrophyllia* sp. and *E*. *fistula*. Hypoxia inducible factor activates the transcription of over 40 genes under low oxygen environments^15^. Also, tauropine dehydrogenase, suggested to play a role in redox balance during hypoxia^16, 17^, was found highly expressed in *Dendrophyllia* sp. and *E*. *fistula*. Significantly increased expression of genes related to hypoxia for *R*. *typus* were limited to a homolog of aryl hydrocarbon receptor nuclear translocator. This protein, in combination with other adaptor proteins, acts as a transcriptional regulator to adaptive hypoxia response^18^. *Dendrophyllia* sp. also showed increased expression of genes related to glycoprotein catabolism, suggestive of high recycling of sugar compounds. Genes involved in vesicle formation and trafficking were upregulated in *E*. *fistula* as well as a homolog of DNA-directed RNA polymerase, which plays a central role in detecting and limiting infection by intracellular bacteria^19^. Additionally, *E*. *fistula* showed high expression of actin-associated genes related to motion, e.g. filopodia, cell motility, and microvilli.

## Discussion

In this study, we conducted transcriptome analyses and gene expression profiling of three deep-sea corals from the Red Sea. Our data revealed that overall the three deep-sea coral transcriptomes seem similar in functional content and all show enrichment for DNA binding, Ca^2+^ binding, and stress response domains (among others) in comparison to cnidarian reference genomes. Further, our analysis of highly expressed genes and differentially expressed orthologs allowed insight into the shared and distinct conditions that govern the physiology of deep-sea corals in the Red Sea, such as warm temperatures, limited organic nutrients, and low oxygen. Interestingly, a recent study that investigated bacterial community composition of the same three deep-sea coral species from the Red Sea, identified anaerobic bacteria and bacteria with the potential to degrade crude oil components as members of the microbiomes^20^. As such, functional adaptions to this unique environment may be reflected by microbial community structure as well as expression of coral genes. The latter can provide insight into the molecular mechanisms employed by the coral host to cope with this particular deep-sea environment and are discussed in the following.

First off, although care was taken to minimize the time between collection and preservation of samples, some genes are known to change expression within minutes (e.g., heat shock proteins). Accordingly, some of the expression patterns observed might come from sample handling and collection. Further, only samples for *E*. *fistula* and *R*. *typus* were preserved *in situ* at depth, but this did not seem to affect the quality of the transcriptome for *Dendrophyllia* sp. in regard to number of assembled and annotated genes or assembly length (contig N50) (Table 1). We tried to minimize potential biases arising from differences in preservation by focusing on highly expressed genes and conducting differential expression analysis based on orthologs (i.e., genes in different species that evolved from a common ancestral gene and are thought to retain the same function in the course of evolution), rather than homologs. Despite similarities in highly expressed genes across species, all species harbored specifically enriched GO terms. Similarly, analysis of expression of orthologs indicated significant expression differences for about 20% of the genes considered. Of note, the closer taxonomic relationship of *Dendrophyllia* sp. and *E*. *fistula* (both family Dendrophylliidae) in comparison to *R*. *typus* (family Flabellidae) did not seem to account for increased similarities in gene expression based on a principal component analysis (Supplemental Information, Figure S1). Taken together, results from highly expressed genes and expression of orthologs shows that all three species at least partly harbor distinct mechanisms to cope with the deep-sea environment of the Red Sea. This suggests that all three species might occupy different ecological niches, which is further supported by prevailing environmental differences between habitats where coral species were collected (Supplemental Information, Table S2). For instance, while temperature was remarkably consistent for all three species (~21.5 °C), oxygen, PON (particulate organic nitrogen), and POC (particulate organic carbon) were markedly different and on average distinctively lower for the environment in which *E*. *fistula* specimens were collected, suggesting that *E*. *fistula* resides in environments with less ability to rely on heterotrophic feeding (Supplemental Information, Table S2).

Despite these differences, however, all corals had genes associated with a function in innate immunity among the highly expressed genes and innate immunity was a significantly enriched GO term for the highly expressed genes in all coral species. The innate immune system can be activated in several ways, but a common denominator is that it is initiated by stimuli that are detected by receptors on the host cell surface. These stimuli activate downstream signaling pathways and ultimately result in a variety of effector responses^21, 22^. One effector response is the production of AMPs (anti-microbial peptides) for antimicrobial defense. In this response, Toll-like receptors (TLRs) activate downstream pathways with a variety of adaptor proteins leading to the expression of NF-κB and its homologs, which in turn induce the production of AMPs^23^. Components of the Toll-signaling pathway have previously been characterized in anthozoans^24^, including the tropical coral *A*. *digitifera* ^25^. In this study, analysis of highly expressed genes revealed that *Dendrophylia* sp. and *R*. *typus* were highly enriched for genes related to TLR signaling pathways. The genes annotated to TLR signaling pathways included transcription factor AP-1 and NF-κB, required for transcriptional activation of immune genes^26^, TNF receptor-associated factor 4, and baculoviral IAP repeat-containing proteins, a regulator of TLRs, as well as several TLRs. Interestingly, we did not identify adaptor proteins of the Toll pathway, which might be explained by low expression of these genes in our data. However, the absence of adaptor proteins was noted in other cnidarians as well, e.g. *H*. *magnipapillata*, *N*. *vectensis*, and *A*. *millepora* and may indicate that these genes are evolutionary lost^13^.

A second immune pathway present in invertebrates is the melanin-synthesis pathway^27^. Though more commonly known for its photo-protective properties, this pathway also confers encapsulation of foreign bodies and cytotoxic defense with the production of reactive oxygen species (ROS)^28^. Tyrosinase-type melanin pathways have been well studied in corals where they were identified in 22 Indo-Pacific hard and soft coral^29^. Functional studies have demonstrated the involvement of this pathway in coral resistance to infection of gram-negative bacteria and fungi^29, 30^. Genes annotated to tyrosinase and syntenin-1, a melanoma differentiation-associated protein were found in the highly expressed gene set for *Dendrophyllia* sp. Additionally, the enriched GO term for melanosome in the highly expressed gene set suggests the importance of this pathway in the immune response of *Dendrophyllia* sp.

Further to the AMP and melanin synthesis pathways, the complement signaling pathway is an innate immunity activation pathway, which uses C-type lectins as signaling receptors that become activated upon binding carbohydrate residues on bacterial cell walls and tissue injury^31^, eventually leading to phagocytosis of the foreign body or cell debris^21^. Complement activation genes, e.g. C-type lectins were among the highly expressed genes in *Dendrophyllia* sp., and C-type lectins have been found in a variety of shallow water coral^32–34^ where they are suggested to play a role in *Symbiodinium* recognition^35, 36^. The presence of complements C2 and C3, downstream of C-type lectin activation, in the highly expressed gene set of *Dendrophyllia* sp. and *E*. *fistula* suggests the involvement of this pathway in the immune response of deep-sea corals. The effector response of the pathway results in phagocytosis, which is supported by the enriched GO terms for lysosome and multivesicular body (i.e. endosome) in *Dendrophyllia* sp. and *E*. *fistula*, respectively.

It is highly interesting to note that innate immune system components commonly associated with the cnidarian-*Symbiodinium* symbiosis in shallow-water corals (e.g., TLRs and C-type lectins)^12, 13, 25, 37, 38^, seem to be equally present in azooxanthellate deep-sea corals. This suggests that these pathways, besides their putative role in the cnidarian-*Symbiodinium* symbiosis, play an essential role in the immunity of all corals. The importance of innate immune pathways to all scleractinian corals might be best highlighted by the presence of Tyrosinase-type melanin pathways that seem ubiquitous in corals and are involved in coral resistance to bacterial and fungal infection, which are arguably important in shallow-water coral reefs and deep-sea environments alike^29, 30^. In support of this, a recent study that characterized bacterial community composition of the same three deep-sea coral species from the Red Sea found species-specific microbiomes supporting a role of innate immune system components in structuring and selecting bacterial microbiomes of deep-sea corals^20^.

Despite common expression of innate immunity-associated genes, deep-sea corals in the Red Sea must contend with several relatively extreme environmental conditions, one of which is low dissolved oxygen (DO) in the water column. DO values are commonly below 2 mg/l at >200 m depth in the Red Sea, while in other seas and oceans where deep-sea coral are found, values range typically from 2.6–6.7 mg/l^4, 6, 39^. However, oxygen limitation within deep-sea corals may be prevalent: Neulinger, *et al*.^40^ found indication for hypoxia in the polyps of *L*. *pertusa*.

Endothermic vertebrates generally employ energy compensation strategies, enhancing oxygen delivery to hypoxic tissues (via vasodilation, increased erythropoiesis, etc.) or in more severe cases by switching to anaerobic glycolysis^41–43^, while the invertebrate strategy to manage low oxygen concentrations is based on energy conservation, i.e. metabolic depression^44–46^. Among the highly expressed genes in *Dendrophyllia sp*. and *E*. *fistula*, glyceraldehyde-3-phosphate dehydrogenase (which catalyzes the first step in glycolysis) is present, while in *R*. *typus* fructose-bisphosphate aldolase, an enzyme playing a role in both gluconeogenesis and glycolysis pathways, was highly expressed. The high expression of these enzymes may suggest that these coral also employ some energy compensation strategies to manage hypoxia.

A study on *Drosophila melanogaster* ^47^, adapted to hypoxic conditions through long-term laboratory selection, demonstrated that mitochondrial metabolism depression and reduction of mitochondrial reactive oxygen species (ROS) production occurred concomitantly with increased activities in complexes I (NADH dehydrogenase) and IV (cytochrome C) of the mitochondrial respiratory chain as well as HIF-1α stabilization by complex III mediation of ROS signals. In the highly expressed gene set of *E*. *fistula*, components of mitochondrial respiratory chain complexes I, III, IV, and V were present, suggesting mitochondrial hypometabolism to manage the low dissolved oxygen concentrations in the deep Red Sea. We found components of complexes III, IV, and V in the highly expressed gene sets of *Dendrophyllia* sp. and of complexes IV and V in *R*. *typus*, while complex II was conspicuously absent across all highly expressed gene sets. We additionally identified HIF-1α, which has been shown to down-regulate mitochondrial oxygen consumption^48^ in the highly expressed gene set of *Dendrophyllia* sp. and *R*. *typus*. In line with this, differential expression of genes involved in glucose transport and associated with aerobic ATP production and hypoxia response were found in shallow-water corals, when investigating coral gene expression differences at noon and midnight in corals from Ofu Island, American Samoa^49^. In particular, the HIF system in shallow-water corals is hypothesized to mediate diel cycles in central metabolism^50^. In this regard, it may be intriguing to obtain daylong expression profiles of deep-sea corals to understand whether a diel cycle exists in the absence of light and endosymbiosis with implications for the evolutionary origin and conservation of these features across stony corals.

Furthermore, a ROS scavenging protein, superoxide dismutase [Cu-Zn], was found in all highly expressed gene sets. The high expression of a variety of hypoxia and hypometabolism-related genes indicates that these corals employ energy conservation strategies to manage the low dissolved oxygen conditions prevalent in the deep Red Sea. This is especially interesting in regard to a recent study on aquaria-reared *E*. *fistula* from the Red Sea^8^, where *E*. *fistula* polyps were shown to increase metabolism at high oxygen levels of more than 8.5 mg/l indicating high phenotypic plasticity. Broad acclimation capacity to prevailing environmental conditions might be one of the reasons explaining the cosmopolitan distribution of *E*. *fistula*. In this context, it would be interesting to assess expression of hypoxia and hypometabolism-related genes at high oxygen levels for *E*. *fistula* and to assess whether phenotypic plasticity is reflected in transcriptional plasticity. This seems to be the case for bacterial communities associated with *E*. *fistula*: a recent comparison of bacterial communities associated with freshly collected *E*. *fistula* from their native Red Sea environment and long-term aquaria reared *E*. *fistula* found largely distinct microbial communities, suggesting that phenotypic plasticity aligns with microbiome flexibility^51^. It remains to be determined, whether this is a general feature of all deep-sea coral species or a characteristic of *E*. *fistula*.

Apart from high temperatures (>20 °C) and low oxygen levels (<2 mg/l), food availability is presumably low in the deep-sea environment of the Red Sea^4^. In line with previous observations^4^, collected specimens of *Dendrophyllia* sp. and *E*. *fistula* showed extreme tissue reduction, e.g. polyps were marginally covered by tissue below the growing edge and different polyps were rarely connected by tissue. Conversely, single-polyp colonies of *R*. *typus* were fleshy, did not show any tissue reductions, and were attached on abandoned sea urchin shells upon collection. Nevertheless, even if deep-sea corals in the Red Sea are specifically adapted to low respiration and calcification rates (in comparison to, e.g. *L*. *pertusa*)^4^, basic metabolic requirements need to be covered. In this regard, it has recently been demonstrated that scleractinian corals can actively facilitate the exchange of amino acids, inorganic carbon, Ca^2+^, and dissolved oxygen by actively stirring water and creating vortices with epidermal cilia^52^. This mechanism can increase mass transfer rates by up to 400% compared to molecular diffusion in an unstirred boundary layer. In our differential gene expression analyses, four genes involved in cilia formation, maintenance, and function were upregulated in *E*. *fistula*. Given the low dissolved oxygen content in the water column surrounding the deep-sea coral, cilia enhanced transport may play a critical role in oxygenation and nutrient transport of coral tissues in *E*. *fistula*. We also found higher expression of the cell division control protein 42 homolog gene in *E*. *fistula*. This protein regulates microtubule attachment to kinetochores, whose components are present in chromosomes as well as in cilia microtubule capping^53^. Further, pleckstrin homology domain containing family M Member 2 that may play a role in regulating kinesin^54^, which is required for cilia assembly^55^, was upregulated as well as genes for alpha and beta tubulin. As transcription of cilia-related genes does not necessitate a cilia-stirred boundary layer, future research should define flow patterns and micrographs of cilia on the coral epidermal surface to determine enhanced mass transport from ciliary beating.

Taken together, while deep-sea reef habitats host hundreds of species and provide nursery grounds for commercially important fish, research is scarce due to difficulty of accessing these environments. In this study we generated reference transcriptomes and conducted *in situ* expression profiling of three deep-sea corals (*Dendrophyllia* sp., *E*. *fistula*, and *R*. *typus*) from the Red Sea to provide baseline data and study the functional attributes that permit these coral to live in deep-sea habitats. Analysis of highly expressed genes suggests that immunological components present in shallow-water corals are also active and present in their deep-sea counterparts, in particular components of the TLR pathway. Further, the expression of genes involved in mitochondrial hypometabolism, anaerobic metabolism, and cilia formation, maintenance, and function offer potential coping mechanisms to low dissolved oxygen concentrations in oligotrophic waters. Future studies should assess which mechanisms represent specific adaptations to the Red Sea environment or can be ascribed to more general strategies of corals living in deep-sea environments.

## Methods

### Study sites and sample collection

Corals in this study were collected by remotely operated vehicle (ROV) during a central and northern Red Sea expedition (KRSE2013L6) from 11–22 May 2013 on the R/V Aegaeo (operated by the Hellenic Center for Marine Research, Greece). Three coral species, *Dendrophyllia* sp. (family Dendrophylliidae), *Eguchipsammia fistula* (family Dendrophylliidae), and *Rhizotrochus typus* (family Flabellidae), were collected with a grabbing basket (Supplemental Information, Figure S2) each with 4 biological replicates for a total of 12 samples. *Dendrophyllia* sp. was collected between 625 and 630 m depth (N22°46.167′, E38°03.102′; KRSE2013L6 ROV dive 17), *E*. *fistula* between 314 and 320 m depth (N22°17.837′, E38°53.811′; KRSE2013L6 ROV dive 07), and *R*. *typus* between 970 and 993 m depth (N27°44.215′, E35°08.000′; KRSE2013L6 ROV dive 13) (Supplemental Information, Tables S1 and S2). A specifically designed two-compartment container was used to place the collected *E*. *fistula* and *R*. *typus* samples into RNAlater at depth for preservation of their *in situ* transcriptomic state (Supplemental Information, Figure S3). The time between collection and placement into RNAlater was between 20 to 45 min for all samples collected. For *Dendrophyllia* sp., samples were collected at depth, placed in a plastic basket, and brought to surface within 90 minutes. Samples from each coral species were collected on separate ROV dives to prevent cross contamination (Supplemental Information, Table S1). On board, coral samples were rinsed with filtered seawater (0.22 µm), crushed in liquid nitrogen, and the pulverized coral samples were stored in cryotubes at −80 °C until RNA extraction.

### RNA isolation and sequencing

RNA from flash frozen coral powder (see above) was isolated using the AllPrep DNA/RNA Mini kit (Qiagen, Hilden, Germany) following the manufacturer’s instructions. mRNA was selected from the total RNA by polyA+ selection using the Dynabead mRNA Purification Kit (Life Technologies, Carlsbad, USA) following the manufacturer’s protocol. PolyA+ selected mRNA, henceforth referred as coral mRNA, was cleaned and concentrated using the RNeasy MinElute cleanup kit (Qiagen). Around 10 ng of coral mRNA were used to generate 180 bp cDNA libraries, which were constructed using the NEBnext Ultra Directional RNA library kit (NEB, Ipswich, USA) following the manufacturer’s protocol. The 12 coral libraries were indexed and sequenced on 1 lane on the Illumina HiSeq platform (San Diego, USA) with 2% phiX non-indexed control at the KAUST sequencing facility.

### Coral transcriptome assembly and gene function enrichment analyses

Adaptor and Illumina-specific sequences were trimmed using Trimmomatic (v. 0.32)^56^ and poor quality portions (Phred < 20) of reads were clipped (4 bp sliding window). Additionally, reads <60 bp in length were discarded. Only paired end reads were retained for the analysis and were error corrected using the standalone error correction module from ALLPATHS-LG (v. 48268)^57, 58^. mRNA sequences from all 4 replicates of a given species were combined for *de novo* transcriptome assembly using Trinity (v. 20131110)^59^ with the following settings: a minimum *k*-mer coverage of 3 and a minimum contig length of 103 bp. The latter condition required a read to assemble, at minimum, with its matched pair. Assembled contigs were filtered using a custom perl script to obtain the longest transcript for each locus (i.e., gene), which was subsequently used as the reference sequence for this gene/locus. A minimum length cutoff of 250 bp was applied and the resulting reference transcriptomes were annotated with SwissProt and TrEMBL UniProt^60^ and with the gene ontology (GO) databases^61^ using a cutoff of 1e-5.

To compare differences between azooxanthellate and zooxanthellate corals and between other cnidarians, we compared Pfam annotations of the three deep-sea coral transcriptomic gene sets (using protein-translated ORFs predicted by TransDecoder)^62^ to the protein-translated genomic gene sets of *Stylophora pistillata* ^63^, *Acropora digitifera* ^25^, *Aiptasia* sp.^64^, *Nematostella vectensis* ^65^, and *Hydra magnipapillata* ^66^. Protein sequences of the three deep-sea coral transcriptomes and of the genomic gene sets were annotated against the PfamA database^67^ using HMMR^68^. Each identified protein domain was tested for enrichment with a Fisher’s exact test applying a Bonferroni corrected *p*-value cutoff of 0.05. Zscore-transformed domain counts were plotted as a heatmap displaying enriched Pfam domains as rows (clustered by Euclidean distance) and organisms as columns using the function heatmap2 from package gplots in R^69^. Assembled transcriptomes and gene annotations are available at http://dsc.reefgenomics.org ^70^.

### Highly expressed genes

Error corrected reads for each library were mapped to the assembled transcriptomes using Bowtie2 (v. 2.1.0)^71^. Subsequently, gene abundance and expression data were calculated in eXpress (v. 1.5.1)^72^. Genes for a given species whose average FPKM values were >100 (Supplemental Information, Dataset S1) were used as input to the R package TopGO^73^ to test for enriched GO terms among highly expressed genes in comparison to a background list of GO terms for all genes from each of the respective species. We used the default topGO “weight01” settings, and considered terms that had a *P* value of <0.05 as significant. The resulting *P* values were not corrected for multiple testing as non-independent tests are carried out on each GO term by topGO^73^.

### Ortholog identification and differentially expressed genes

To compare gene expression between coral species, orthologs of the three coral transcriptomes were identified based on a reciprocal BLAST approach. TransDecoder (v. 20120815)^62^ was used to identify open reading frames (ORFs) and pairwise orthologs were identified on the amino acid level using InParanoid (v. 4.1)^74^. MultiParanoid^75^ was used for multispecies assignment of orthologs. In an effort to reduce spurious assignments, only pairwise InParanoid groups with a bit score >300 were used as input for MultiParanoid^75^. Additionally, only ortholog groups comprising of exactly 3 genes were retained in the analysis (i.e. each coral species contributed exactly one gene to an ortholog group and ortholog groups with paralogs were not considered). FPKM values for orthologs from each coral species were retrieved from eXpress (see above) (Supplemental Information, Dataset S2). To compare expression of orthologs across all species, a principal coordinate analysis (PCA) was conducted in MeV^76^. FPKM values of orthologs were log_2_(x + 1) transformed before statistical testing. Differentially expressed orthologs were identified via a one-way ANOVA using FDR < 0.05 obtained from QVALUE (R package)^77^ (Supplemental Information, Dataset S3). Differentially expressed orthologs were clustered based on a 0.7 distance threshold under the Pearson correlation distance metric using the complete linkage method in MeV^76^.

### Accession codes

Sequence data determined in this study are available at NCBI’s BioProject database under PRJNA275034 (http://www.ncbi.nlm.nih.gov/bioproject/275034, *Dendrophylia* sp.), PRJNA275035 (http://www.ncbi.nlm.nih.gov/bioproject/275035, *E*. *fistula*), and PRJNA275037 (http://www.ncbi.nlm.nih.gov/bioproject/275037, *R*. *typus*). Assembled transcriptomes and gene annotations are available at http://dsc.reefgenomics.org.

## Electronic supplementary material

Supplementary Information

Dataset S1

Dataset S2

Dataset S3
