# Supplementary material for: Transcriptomes and expression profiling of deep-sea corals from the Red Sea provide insight into the biology of azooxanthellate corals
Source: Sci Rep. 2017 Jul 25;7:6442. doi: 10.1038/s41598-017-05572-x (PMC5526985; doi:10.1038/s41598-017-05572-x)
Supplement: Supplementary file 1 — Supplementary Information [file 41598_2017_5572_MOESM1_ESM.pdf]

Transcriptomes and expression profiling of deep-sea corals from the Red Sea provide insight into the biology of azooxanthellate corals

Lauren K. Yum[1], Sebastian Baumgarten[1], Till Röthig[1], Cornelia Roder[1], Anna Roik[1], Craig Michell[1], Christian R Voolstra[1,*]

[1]Red Sea Research Center, Division of Biological and Environmental Science and Engineering (BESE), King Abdullah University of Science and Technology (KAUST), Thuwal 23955-6900, Saudi Arabia

*Corresponding Author:

Christian R Voolstra

Email: christian.voolstra@kaust.edu.sa

Phone: +966218082377

Keywords: Red Sea, deep-sea coral, transcriptome, azooxanthellate corals, cold-water coral, expression profiling

Running title: Red Sea deep-sea coral transcriptomes

Supplemental Information

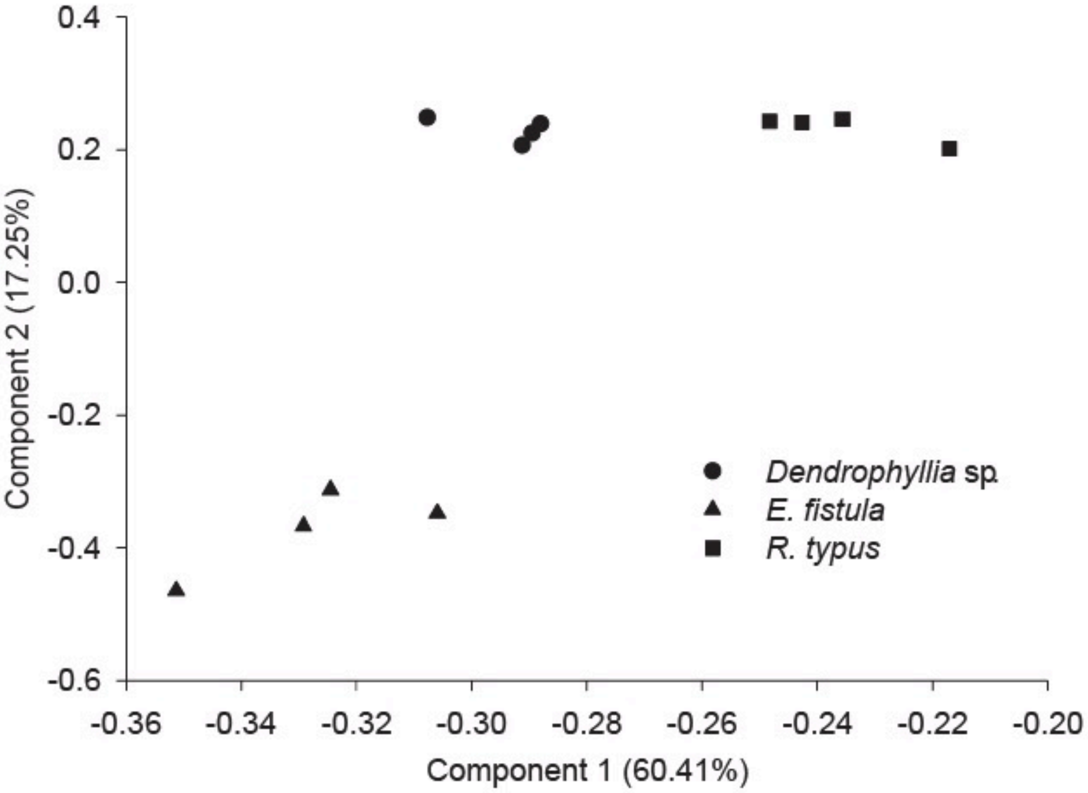

Figure S1. Principal component analysis (PCA) plot based on the expression of 2,069 orthologous genes. Clustering is by coral species and explains the majority of variation.

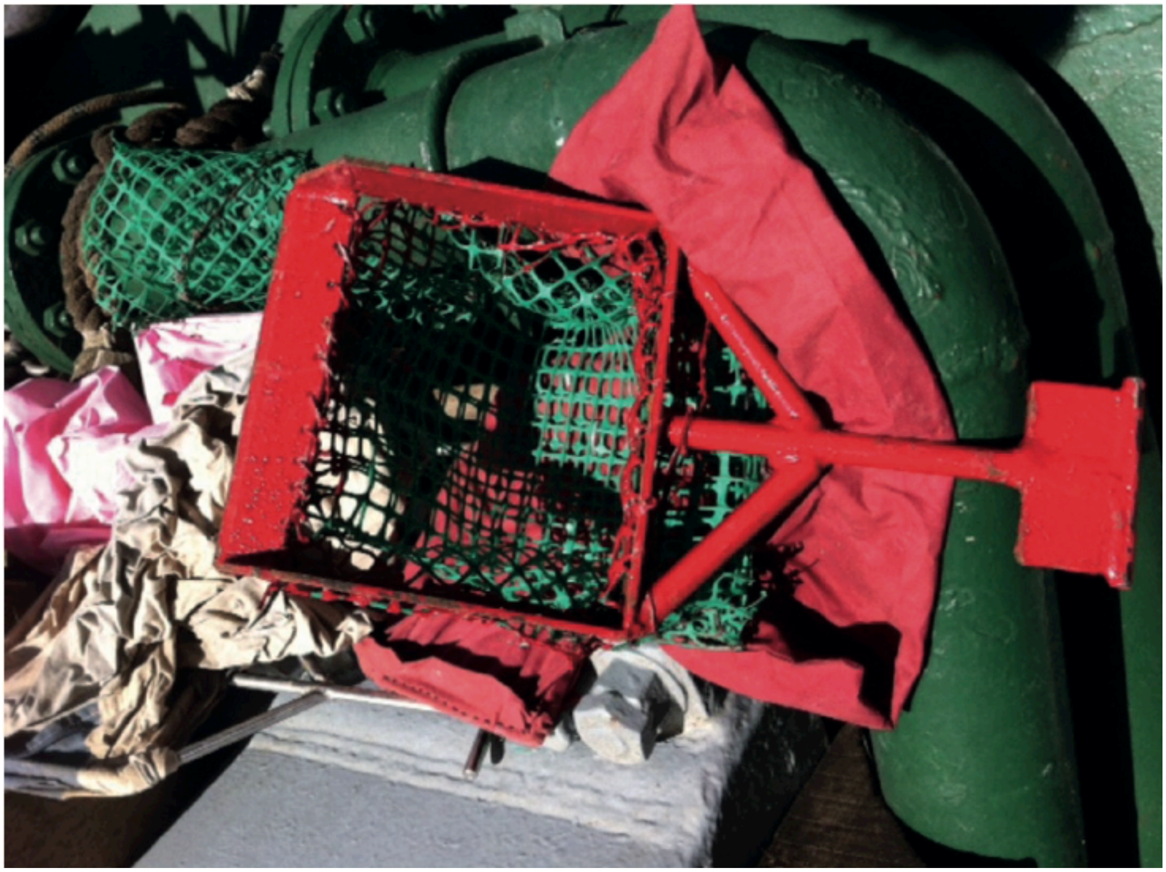

Figure S2. Grabbing basket used to collect deep-sea coral samples.

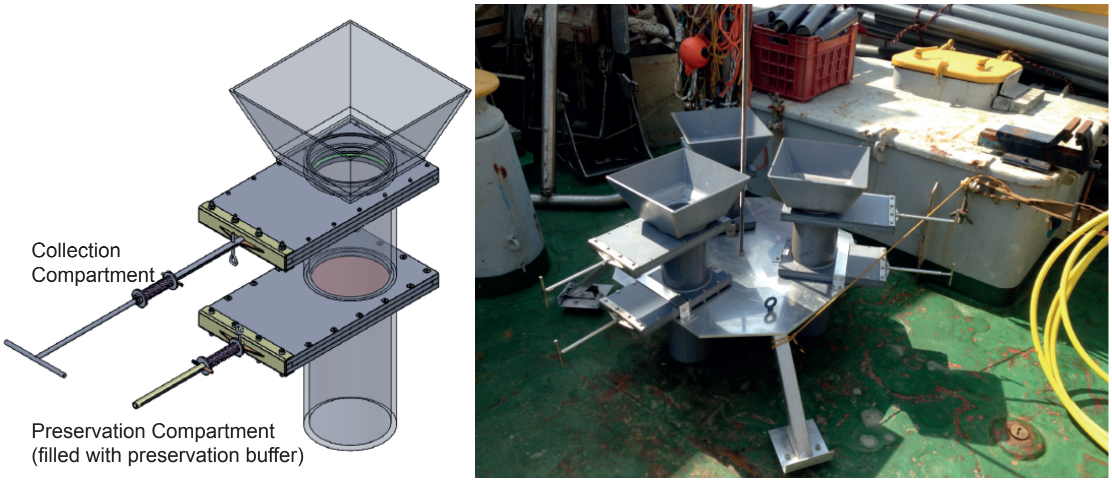

Figure S3. Sample collection container. The collection container consists of two compartments that can be independently opened and closed. Initially, both compartments are closed and the preservation compartment is filled with preservation buffer. Upon sample collection, the collection compartment is open and samples are retained in this compartment. At the end of sample collection, the collection compartment will be closed and the preservation compartment will be opened. This results in samples falling into the preservation buffer. The preservation compartment is closed and samples are transported back to the ship where they can be retrieved.

Table S1. Sample collection details for ROV dives.

Species	Date	ROV dive number	Start time	End time	Min depth (m)	Max depth (m)	Latitude start	Latitude end	Longitude start	Longitude end
Dendrophyllia sp.	22-May-13	17	15:57	17:02	625	630	22˚46.149	22˚46.167	38˚02.944	38˚03.102
E. fistula	11-May-13	07	09:43	11:45	320	314	22˚17.792	22˚17.837	38˚53.717	38˚53.811
R. typus	18-May-13	13	15:47	17:21	970	993	27˚44.225	27˚44.215	35˚07.006	35˚08.000

Table S2. Environmental parameters of habitats where corals samples were collected. Parameters depth, temperature, pressure, and oxygen measured by CTD casts (±5m bins were averaged around listed depths). Remaining parameters measured by ROV-collected water in Niskin bottles. PON, particulate organic nitrogen (>0.7 µm); POC, particulate organic carbon (>0.7 µm).

**Species**	**Depth [m]**	**Temp [°C]**	**Pressure [dbar]**	**Oxygen [mg/l]**	**pH**	**PON/l [µg]**	**POC/l [µg]**	**Phosphate [µM]**	**Silicate [µM]**	**Nitrite/ Nitrate [µM]**	**Ammonia [µM]**
Dendrophyllia sp.	597	21.58	602.37	2.08	7.94	4.83	41.33	0.88	14.65	19.79	0.91
E. fistula	359	21.61	361.48	0.53	8.31	2.53	27.43	0.97	13.19	21.08	0.4
R. typus	982	21.65	969.72	3.4	8.01	4.22	39.48	0.62	8.96	14.77	0.8

Dataset S1. Genes of the deep-sea corals *Dendrophylia* sp. (n = 832), *E. fistula* (n = 750), and *R. typus* (n = 466) with average FPKM values >100.

Dataset S2. Orthologs of *Dendrophylia* sp., *E. fistula*, *R. typus* and associated expression values (FPKM) over colony replicates.

Dataset S3. Differentially expressed orthologs and associated $\log_2(x+1)$ FPKM values for all samples.
